# Prevalence and type distribution of human papillomavirus infection in women from North Sardinia, Italy

**DOI:** 10.1186/1471-2458-11-785

**Published:** 2011-10-11

**Authors:** Andrea Piana, Giovanni Sotgiu, Paolo Castiglia, Stefania Pischedda, Clementina Cocuzza, Giampiero Capobianco, Vincenzo Marras, Salvatore Dessole, Elena Muresu

**Affiliations:** 1 Department of Biomedical Science, Hygiene and Preventive Medicine, University of Sassari, Sassari, Italy; 2 Department of Clinical Medicine and Prevention, University of Milano-Bicocca, Italy; 3 Gynecologic and Obstetric Clinic, University of Sassari, Sassari, Italy; 4 Department of Surgical Pathology, University of Sassari, Sassari, Italy

## Abstract

**Background:**

Human papillomavirus (HPV) has been associated with several disorders of the genital tract, skin and oropharynx. The aims of our study were to evaluate the prevalence of HPV infection in women between 15 and 54 years of age in North Sardinia, Italy, to identify the prevalence of High Risk - Human papillomaviruses (HR-HPV) genotypes and to establish a correlation between molecular and cytological results.

**Methods:**

From 2007 to 2009 we consecutively enrolled women aged 15-54 years admitted to public and private outpatient settings. All the participants filled in a questionnaire about the socio-cultural state, sexual activity and awareness about HPV. 323 cervical specimens were tested for HPV-DNA and HPV genotypes with INNO-LiPA HPV Genotyping CE Amp kit. Samples showing positivity to some HPV genotypes were re-tested using "in house" quantitative Real-Time PCR assays.

**Results:**

Overall HPV-DNA positivity was detected in 35.9% of the women. The prevalence of HR-HPV infection among HPV positive samples was 93.1% with a specific prevalence of HPV 16, 51, 31, 53 and 18 of 54.3%, 37.9%, 10.3%, 6.9% and 5.2%, respectively. Co-infection with any HPV, HR-HPV, LR-HPV and HR/LR-HPV type was 18.3%, 14.9%, 0.9% and 2.5%, respectively; HPV 16/51 co-infection was detected in 64.6% of the HR-HPV co-infection group. The most frequent HPV-genotypes detected were 16 (32.5%) and 51 (22.7%). Among the 57 patients harboring mono-infection the most prevalent HPV genotypes were 16 (38.6%) and 31(10.5%). A multivariate analysis identified a statistical significant association between HPV infection and age and between HPV infection and previous sexual transmitted diseases. A statistically significant association between cytological cervical lesions and generic HPV exposure was identified.

**Conclusions:**

To our knowledge, this is the first survey evaluating the prevalence of HPV infection in Northern Sardinia and drawing attention to the unusual high proportion of genotype HPV 51. Given the recent implementation of a widespread immunization program with vaccines not containing HPV 51, it has been relevant to prove the high prevalence of this HPV genotype from the start of the vaccination campaign, in order to avoid in the future attributing to the vaccination program a possible selection effect (HPV replacement).

## Background

Human papillomaviruses (HPVs), double stranded DNA viruses belonging to family *papillomaviridae*, are highly epitheliotropic viruses and have been associated with various cutaneous or mucosal clinical manifestations ranging from benign hyperplastic epithelial proliferative innocuous lesions (warts, papillomas) to cancer of the genital tract, skin, oropharynx [1,2]; in particular, they have been recognized as causative agents in the development of cervical cancer [3].

Several genotypes are defined as high-risk (HR-HPV) being associated with a comparatively high risk for invasive neoplasia; furthermore, they are sub-classified as carcinogenic (types 16, 18, 31, 33, 35, 39, 45, 51, 52, 56, 58 and 59), probably carcinogenic (type 68) and possibly carcinogenic (types 26, 30, 34, 53, 66, 67, 69, 70, 73, 82, 85 and 97) to humans [4].

HPV types 16 and 18 account for approximately 70% of cervical cancer cases worldwide with other high-risk types such as HPV-45, HPV-31, HPV-33 and HPV-52 accounting for the majority of the remaining cervical malignancies [5]. HPV "low-risk" types (LR-HPV), mainly HPV-6 and HPV-11, are rarely detected in high-grade cervical lesions but cause the majority of anogenital warts [6].

The link between HPV infection and cervical cancer has given impetus to the development of prophylactic vaccines against the most common HR-HPV types; in parallel, interest has been raised to determine age specific burden of HPV infection and to identify the major genotypes supporting infection and diseases in different countries.

Heterogeneous distribution of HPV prevalence and genotypes has been described worldwide [7].

In Italy, a recent cross-sectional study on HR-HPV infection showed an overall prevalence of 14.8% countrywide, without significant differences among geographical regions [8].

However, another recent study [9] carried out in the South of Sardinia, Italy, showed that 31% of the tested women were HPV-positive; single or multiple infections sustained by HPV-16 or HPV-18 represented 43.5% of all HPV infections.

Furthermore, another interesting finding from previous Italian studies was the relatively high prevalence observed of HPV-51 [8,10-12]. It is expected that the implementation and scale-up of the national immunization HPV strategy, by the use of one of the two commercially available vaccines, Cervarix™ and Gardasil^® ^in adolescents aged 12 years since 2007 could modify the epidemiological scenario.

The aims of our study were to evaluate the prevalence of HPV infection in women between 15 and 54 years of age in North Sardinia, Italy, to identify the prevalence of HR-HPV genotypes and to establish a correlation between molecular and cytological results.

## Methods

### Study population

In Sardinia, Italy, the average coverage of PAP-test in women aged 25-64 years is 49.3%, of which 24.9% on voluntary basis [13,14]. During the study period (November 2007 - January 2009) we consecutively enrolled women aged between 15 and 54 years admitted to public and private outpatient settings located in the town of Sassari, Northern area of Sardinia, where cervical screening, unlike other Italian geographical areas, was performed on a voluntary basis and not as part of a prevention program [14-17].

Personal data have been treated in compliance with the Law Decree No. 196/2003, article 24 (Code for the protection of personal data). This study, for which a written consent from each enrolled patient or her relatives was obtained, was formerly approved by the Ethical Committee of the Azienda Sanitaria Locale n°1 of Sassari (PN-132) on June 18, 2007.

### Statistical analysis

A power of 80% and a level of statistical significance of 5% was necessary to determine the sample size in order to detect a difference between proportions: on the basis of the female population aged 15-54 in Sassari in 2006 (N = 37,353 women), the eligible (i.e., 24.9% of the total referring to specialized centers on a voluntary basis) population was 9,301 women. Since the prevalences of HPV-positivity and of HPV-16, estimated in an Italian city, (8.8% and 2.87%, respectively [22]), the overall sample size for any HPV infection was 318 individuals and the sample size for each planned age group (i.e., 15-24, 25-34, 35-44, 45-54 years) for the most frequent HPV type (i.e., 2.87% for HPV16) was 43. Therefore, at least 50 individuals were expected to be enrolled in every group.

Difference between proportions were compared via z-test. Categorical variables were compared by Chi-squared test and continuous variables by t-test or Mann-Whitney test. Logistic regression analysis of the association between HPV infection in cervical lesions with potential covariates was performed.

A p-value of ≤0.05 was considered statistically significant. Data were collected on standardised e-forms and analysed using Stata 9.0 (StataCorp, Stata Statistical Software Release 9, College Station, TX, USA, 2005).

### Sample collection

Cervical specimens were collected with Cervex-brush and suspended in a 20 ml preservation solution, PreservCyt transport medium (ThinPrep Pap Test; Cytyc Corporation, Boxborough, Mass.).

### INNO-Lipa HPV Genotyping test (Innogenetics)

The DNA extraction was performed following the protocol described in the High Pure Viral Nucleic Acid kit from Roche Diagnostics. HPV DNA amplification was carried out with INNO-LiPA HPV Genotyping CE Amp kit [18], which targets a 65-bp fragment of the L1 open reading frame; in particular, DNA is PCR amplified using biotinylated primers, deoxynucleotide triphosphates (dNTPs), thermostable DNA polymerases.

The sample mixture is heated in order to separate the two strands of the DNA helix and expose the target sequences to the primers. After 40 cycles, a multi-amplified biotinylated target sequence is obtained.

HPV genotyping was performed using reverse hybridization by the INNO-line probe assay (INNO-LiPA HPV Genotyping Extra). The diagnostic test contains probes for 28 anogenital HPV genotypes (HPV 6, 11, 16, 18, 26, 31, 33, 35, 39, 40, 43, 44, 45, 51, 52, 53, 54, 56, 58, 59, 66, 68, 69, 70, 71, 73, 74, 82). The hybridization steps were carried out following the INNO-LiPA kit instruction. The oligonucleotide probes, together with 3 controls, were immobilized in parallel lines on nitrocellulose membrane strips by the supplier. After hybridization of the PCR product to the probes on the strip under stringent conditions, followed by stringent washing, the hybrids were detected by alkaline phosphatase/streptavidin conjugate; reaction with a substrate (5-bromo-4-chloro-3-indolylphosphate and nitroblue tetrazolium) results in a purple precipitate at the positive probe lines. After drying, the strips were interpreted visually by using the INNO-LiPA HPV genotyping v2 interpretation chart.

Samples found to be positive for HR-HPV genotypes 16, 18, 31, 45, 51 and 52 by INNO-LiPA were further confirmed by the previously described "in-house" Real-Time quantitative TaqMan PCR assays [19-21]. Amplification was performed using using TaqMan technology and ABI Prism device (7900 SDS; Applied Biosystems, Forster City, CA). All reactions were optimized to obtain the best amplification kinetics under the same cycling conditions (denaturation at 95°C for 15 min, followed by 40 cycles of denaturation at 95°C for 15 s and annealing/extension at 60°C for 60 s) and composition of the reaction mixture. Detection and quantification of CCR5 was used to quantify human genomic DNA in each sample and to normalize the viral load [20] and viral load expressed as copies number for 10^4 ^cells.

## Results

The characteristics of the population studied are described in table 1. From November 2007 to January 2009, 323 cytological samples were collected from women whose age ranged from 15-54 years (median; InterQuartile Range: 37; 28-44). The age of the majority of enrolled individuals (199/323, 61.6%) ranged from 25 to 44 years; most of them were Caucasians (309/323, 95.7%), with a high educational level (high school or academic degree: 244/322, 75.8%).

**Table 1:** Epidemiological and clinical characteristics of the enrolled individuals.

Characteristics		Total sample	HPV- individuals	HPV+ individuals	HR/HPV+ individuals
Median (IQR) age		**37 (28-44)**	**38 (30-45)**	32 (25-39)**	**32 (24-38.5)**
Age, n (%)	***15-24***	**52 (16.1)**	**24 (11.6)**	28 (24.1)**	**28 (25.9)**
	***25-34***	**87 (26.9)**	**51 (24.6)**	**36 (31.0)**	**31 (28.7)**
	***35-44***	**112 (34.7)**	**76 (36.7)**	**36 (31.0)**	**35 (32.4)**
	***45-54***	**72 (21.3)**	**56 (27.1)**	16 (13.8)**	**14 (13.0)**
Educational level, n (%)	***Primary school***	**5 (1.6)**	**5 (2.4)**	**-**	**-**
	***Middle school***	**73 (22.7)**	**47 (22.7)**	**26 (22.6)**	**23 (21.5)**
	***High school***	**160 (49.7)**	**95 (45.9)**	**65 (56.5)**	**61 (57.0)**
	***University***	**84 (26.1)**	**60 (29.0)**	**24 (20.9)**	**23 (21.5)**
Married or divorced, n (%)		**187 (58.1)**	**133 (64.6)**	54 (46.6)**	**51 (47.2)**
Smoking habit, n (%)	***Smoker***	**88 (27.3)**	**53 (25.7)**	**35 (30.4)**	**33 (30.8)**
	***Non smoker***	**160 (49.7)**	**104 (50.5)**	**56 (48.7)**	**52 (48.6)**
	***Ex smoker***	**73 (22.7)**	**49 (23.8)**	**24 (20.9)**	**22 (20.6)**
Nulliparity, n (%)		**177 (55.0)**	**102 (49.5)**	75 (64.7)**	**70 (64.8)**
Oral contraceptive use, n (%)		**252 (78.5)**	**155 (75.6)**	**97 (84.4)**	**90 (84.1)**
Mean (SD) age at first sexual intercourse		**18.7 (3.3)**	**19.1 (3.4)**	18.0 (2.9)**	**18.0 (2.9)**
No of partners since first sexual intercourse, n (%)	***0***	**4 (1.2)**	**4 (1.9)**	**-**	**-**
	***1***	**112 (34.8)**	**76 (36.9)**	**36 (31.0)**	**35 (32.4)**
	***2-3***	**121 (37.6)**	**81 (39.3)**	**40 (34.5)**	**37 (34.3)**
	***4-9***	**62 (19.3)**	**31 (15.1)**	31 (27.7)*	**28 (25.9)**
	***> 9***	**23 (7.1)**	**14 (6.8)**	**9 (7.8)**	**8 (7.4)**
No of new partners in the last 3 months, n (%)	***0***	**282 (87.6)**	**189 (91.8)**	93 (80.2)**	**86 (79.6)**
	***1***	**34 (10.6)**	**13 (6.3)**	21 (18.1)**	**20 (18.5)**
	***> 1***	**6 (1.9)**	**4 (1.9)**	**2 (1.7)**	**2 (1.9)**
Use of condom, n (%)	***Never ***	**89 (27.8)**	**55 (26.8)**	**34 (30.1)**	**33 (31.4)**
	***Past ***	**59 (18.4)**	**37 (18.1)**	**22 (19.5)**	**19 (18.1)**
	***Current***	**170 (53.1)**	**113 (55.1)**	**57 (50.4)**	**53 (50.5)**
History of Sexually Transmitted Diseases, n (%)		**56 (17.7)**	**25 (12.4)**	31 (27.4)**	**27 (25.7)**
Compliance to PAP test in the last 3 years, n (%)		**191 (60.1)**	**119 (57.8)**	**72 (64.3)**	**67 (64.4)**
Sample, n (%; 95% CIs)		**323**	**207 (64.1; 57.6-70.6)**	**116 (35.9; 30.7-41.1)**	**108 (33.4; 28.3-38.5)**

IQR: InterQuartile Range

SD: Standard Deviation

CIs: Confidence Intervals

Comparison vs HPV - individuals: *p < 0.05, **p < 0.01

More than half of the enrolled women (57.9%) were married (165 currently married while 22 in the recent past), 50% were smokers and/or ex-smokers, 55.0% (177/323) were nulliparous, most of them taking oral contraceptives. Mean (SD) age of first sexual intercourse was 18.7 (3.3) years.

Number of lifetime sexual partners was at least 2 in more than 50%: 37.6% (121/323) and 19.3% (62/323) had had 2-3 and 4-9 sexual partners, respectively; 10.5% (34/323) had had a new sexual partner in the last three months.

Twenty-eight percent (89/318) of the enrolled individuals stated that they had never used condom during their sexual relationships; 17.8% (56/315) had been affected by sexually transmitted diseases: about half of them being infected by HR-HPV genotypes (27/56, 48.2%) versus about one-third of those without a history of sexually transmitted diseases (78/259, 30.1%).

The proportion of women (191/318, 60.1%), who had had a Pap test in the last three years, was significantly higher than the national average (39.8%) [15-17].

Table 2 reports the prevalence of high and low risk HPV types stratified by age groups and cytological results. The prevalence of HPV infection was 35.9% (116/323), although it showed a significant age-related decrease (53.9%, 41.4%, 32.1% and 22.2% in those whose age ranged from 15-24, 25-34, 35-44 and 45-54 years, respectively. Slope -0.10, P for trend < 0.001); a similar trend was observed for those infected by HR-genotypes: prevalence of HR-HPV infection was 53.9% between 15-24 years, 35.6% between 25-34 years, 31.3% between 35-44 years and 19.4% in those aged more than 44 years (Slope -0.10, P for trend < 0.001).

**Table 2:** Prevalence of high- and low-risk HPV types stratified by age groups and cytological results.

		HPV 16		HPV 18		HPV 51		Other than HPV 16 and 51 HR types		Nearest non-vaccine types (31, 33, 45, 52, 58)		HR types		LR types		Any HPV type		Mono-infection		Co-infection with HR and/or LR types	
		n (%)	95% CIs	n (%)	95% CIs	n (%)	95% CIs	n (%)	95% CIs	n (%)	95% CIs	n (%)	95% CIs	n (%)	95% CIs	n (%)	95% CIs	n (%)	95% CIs	n (%)	95% CIs
Age	**15-24**	**11 (21.2)**	**10.1-32.3**	**5** **(9.6)**	**1.6-17.6**	**7 (13.5)**	**4.2-22.8**	**15 (28.9)**	**16.6-41.2**	**7 (31.8)**	**12.3-51.3**	**28 (53.9)**	**40.4-67.5**	**10 (19.2)**	**8.3-29.7**	**28** **(53.9)**	**40.4-67.5**	**12 (23.1)**	**11.7-34.6**	**16 (30.8)**	**18.3-43.4**
	**25-34**	**19 (21.8)**	**13.1-30.5**	**1** **(1.2)**	**-1.1-3.5**	**11 (12.6)**	**5.6-19.6**	**16 (18.4)**	**10.3-26.5**	**8 (36.4)**	**16.3-56.5**	**31** **(35.6)**	**25.5-45.7**	**10 (11.5)**	**4.4-17.6**	**36** **(41.4)**	**31.1-51.8**	**20 (23.0)**	**14.2-31.8**	**16 (18.4)**	**10.3-26.5**
	**35-44**	**25 (22.3)**	**14.6-30.0**	**0** **(0.0)**	**0.0-0.0**	**20 (17.9)**	**10.8-25.0**	**7 (6.3)**	**1.8-10.8**	**5 (22.7)**	**5.2-40.2**	**35 (31.3)**	**22.7-39.9**	**5 (4.5)**	**0.6-8.2**	**36** **(32.1)**	**23.5-40.8**	**16 (14.3)**	**10.8-25.0**	**20 (17.9)**	**10.8-25.0**
	**45-54**	**8 (11.3)**	**4.0-18.6**	**0** **(0.0)**	**0.0-0.0**	**6 (8.3)**	**2.0-15.0**	**7 (9.7)**	**2.9-16.5**	**2 (9.1)**	**-2.9-21.1**	**14 (19.4)**	**10.3-28.5**	**3 (4.2)**	**0.2-10.8**	**16** **(22.2)**	**12.6-31.8**	**9 (12.5)**	**2.9-16.5**	**7 (9.7)**	**2.9-16.5**
PAP-test	**Normal**	**48 (19.9)**	**4.9-49.0**	**3** **(1.2)**	**-0.2-2.6**	**32 (13.2)**	**8.9-17.5**	**21** **(8.7)**	**5.2-12.3**	**13 (5.4)**	**2.6-8.3**	**84 (34.7)**	**28.7-40.7**	**18 (7.4)**	**4.2-10.8**	**78 (32.2)**	**26.3-38.1**	**42 (17.4)**	**12.6-22.2**	**41 (16.9)**	**12.2-21.6**
	**ASCUS**	**4 (12.5)**	**1.0-24.0**	**2** **(6.3)**	**-2.1-14.7**	**2 (6.3)**	**-2.1-14.7**	**9 (28.1)**	**12.4-43.6**	**4 (12.5)**	**1.0-24.0**	**9 (28.1)**	**12.5-43.7**	**5 (15.6)**	**2.6-27.4**	**14 (43.8)**	**26.6-61.0**	**5 (15.6)**	**3.0-28.2**	**7 (21.9)**	**7.6-36.2**
	**LSIL**	**6 (24.0)**	**7.3-40.7**	**1** **(4.0)**	**-3.7-11.7**	**4 (16.0)**	**1.6-30.4**	**4 (16.0)**	**1.6-30.4**	**3 (12.0)**	**0.7-24.7**	**9 (36.0)**	**17.2-54.8**	**5 (20)**	**4.3-35.7**	**13 (52.0)**	**32.4-71.6**	**6 (24.0)**	**7.3-40.7**	**7 (28.0)**	**10.4-45.6**
	**HSIL**	**2 (50.0)**	**1.0-99.0**	**0** **(0.0)**	**0.0-0.0**	**2 (50.0)**	**1.0-99.0**	**1 (25.0)**	**-17.4-67.4**	**1 (25.0)**	**-17.4-67.4**	**3** **(75.0)**	**32.5-100.0**	**0 (0.0)**	**0.0-0.0**	**4 (100.0)**	**1-1**	**1 (25.0)**	**-17.4-67.4**	**2 (50.0)**	**1.0-99.0**
All screened sample		**63 (19.5)**	**15.3-23.9**	**6** **(1.9)**	**0.4-3.4**	**44 (13.6)**	**9.9-17.5**	**45 (13.9)**	**10.2-17.8**	**22 (6.8)**	**-3.7-17.4**	**108 (33.4)**	**28.3-38.5**	**28 (8.7)**	**5.6-11.8**	**116** **(35.9)**	**30.7-41.1**	**57 (17.7)**	**13.5-21.9**	**59 (18.3)**	**13.8-22.2**

CIs: Confidence Intervals

ASCUS: Atypical Squamous Cells of Undetermined Significance

LSIL: Low-grade Squamous Intra-epithelial Lesion

HSIL: High-grade Squamous Intra-epithelial Lesion

HR: High-Risk genotype

LR: Low-Risk genotype

**Figure 1:**
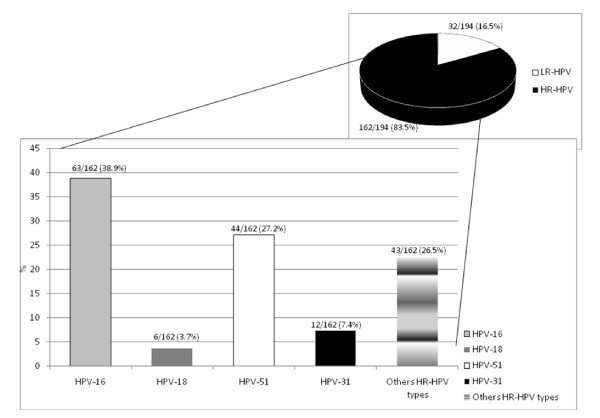
**HPV genotype distribution in the Sardinian cohort**.

From the 116 HPV positive specimens, 194 HPV genotypes were detected (Figure 1): 162/194 (83.5%) HR-HPV genotypes and 32/194 (16.5%) LR-HPV genotypes.

Overall, the most frequent HPV-genotypes detected were 16 (63/194, 32.5%), 51 (44/194, 22.7%), 6 (15/194, 7.7%), 31 (12/194, 6.2%) and 53 (8/194, 4.1%).

Out of 323 women, 108 (33.4%) were positive for at least one HR-HPV genotype. The prevalence of HR-HPV infection among the HPV positive samples was 93.1% (108/116) with a specific prevalence of HPV 16, 51, 31, 53 and 18 of 54.3% (63/116), 37.9% (44/116), 10.3% (12/116), 6.9% (8/116) and 5.2% (6/116), respectively. The most frequent LR-HPV genotypes among the HPV positive samples were type 6 (15/116, 12.9%) and type 11 (6/116, 5.2%).

Co-infection with any HPV, HR-HPV, LR-HPV and HR/LR-HPV type was detected in 59/323 (18.3%), 48/323 (14.9%), 3/323 (0.9%) and 8/323 (2.5%), respectively; HPV 16/51 co-infection was detected in 64.6% of the HR-HPV co-infection group. Overall, the prevalence of the two most frequent types (*i.e*., HPV16 and 51) was 19.8% and 13.8%, respectively. The observed proportion of HP16/51 co-infection was 9.7%, whereas the expected one was 2.7% (p < 0.001). No HPV16/18 co-infection was detected.

Among the 57 patients harboring mono-infection the most prevalent HPV genotypes were 16 (38.6%), 31 (10.5%), 53 (8.8%) and 51 (7.0%).

The specificity of INNO-LiPA positive samples for HR-HPV genotypes 16, 18, 31, 45, 51 and 52 was further confirmed by previously described "in-house" Real-Time quantitative TaqMan PCR assays [19-21]. All retested positive samples were confirmed to harbour the HR-HPV type detected by INNO-LiPA, indicating a very good specificity in genotype detection of this assay. Furthermore overall viral loads detected by the quantitative "in-house" Real-Time PCR assays for the studied HR-HPVs were found to range from 1 to 34750 copies/10^4 ^cells with median values for the two most prevalent HPV genotypes 16 and 51 being 962 and 2055 copies/10^4 ^cells respectively.

Out of 303 women with cytological assessment, 242 (79.9%) showed negative cytology while 32 (10.6%), 25 (8.3%) and 4 (1.3%) showed a pattern of atypical cells of undetermined significance (ASCUS), a low-grade of squamous intraepithelial lesion (LSIL) and a high-grade of squamous intraepithelial lesion (HSIL), respectively (Table 2).

Univariate logistic regression analysis showed that HPV infection was significantly associated with previous sexual transmitted diseases (OR: 2.68; P = 0.001; 95% CI: 1.48 - 4.82), number of sexual partners (OR: 1.30; P: 0.033; 95% CI: 1.02 - 1.66), number of sexual partners in the last three months (OR: 1.98; P: 0.018; 95% CI: 1.13 - 3.47), age (OR: 0.95; p < 0.001; 95% CI: 0.93 - 0.98) and onset of sexual activity (OR: 0.89; P = 0.005; 95% CI: 0.82 - 0.97), whereas, the multivariate analysis identified a statistical significance only for age and previous sexually transmitted diseases.

Although a statistically significant association between cytological cervical lesions and generic HPV exposure (OR: 2.77; P = 0.010; 95% CI: 1.27 - 6.05) was identified, this was not confirmed for infections due to HPV 16, 18 and 51.

## Discussion

Our results highlight that the prevalence of HPV cervical infection in Northern Sardinia (35.9%) is almost similar to that estimated in other Italian geographical areas (33% and 31% in Puglia region and in the province of Cagliari, respectively) [9,22], but higher than that found in other parts [23]. It was recognized an age-related decrease of HPV prevalence, mostly evident in those subjects infected by HR-HPV genotypes.

One of the striking findings is the high prevalence of HPV 16/51 coinfections; our study was aimed at studying HPV infection's prevalence, although it would have been interesting to follow-up co-infected individuals in order to assess the association between cervical neoplasm and multiple infections.

A high frequency of HPV-16 infection, followed by HPV-51 infection, was observed in our survey, whereas the prevalence of HPV-18 infection is significantly lower than that identified in the Southern Sardinian areas [9].

It is remarkable the observed high relative frequency of HPV-51 infection together with other HR- and LR-HPV types: this could be an epidemiological feature of Northern Sardinia, confirming data described in several recent Italian cross-sectional studies (12% -15.3%) where it is frequently detected with other HR-HPV types and in patients affected by CIN 2 [10].

Logistic regression analysis showed a statistically significant association between cytological cervical lesions and HPV infection (OR: 2.77; p = 0.010), but such association was not confirmed with HPV 16, 18 and 51 infections, probably due to the low statistical power of the study, that was set up using the estimated prevalence of HPV-16 infection.

Several variables have been found to be significant associated with HPV infection both positively (previous sexually transmitted diseases, number of sexual partners, number of sexual partners in the last three months) and negatively (age, onset of sexual activity), confirming the validity of the immunization strategy, mainly aimed at girls aged 12 years, before starting of sexual activity.

The successful nationwide implementation of the HPV vaccination program in Italy will probably change the frequency of the most prevalent genotypes, decreasing the prevalence of the current genotypes and increasing the possibility of HPV replacement following vaccination, as already described for several bacterial infections [24]. Even if the probability of occurrence of that condition was deemed low until now [25], it was recently observed a competitive advantage of some HPV genotypes over other genital HPV types in the unvaccinated population [26]. Therefore, HPV genotype prevalence should be monitored for type replacement before, during and after mass vaccination. Surveillance of HPV infections through molecular methods could represent an important mean for the development of new primary and secondary prevention strategies, such as new vaccines targeted to genotypes which might replace those previously prevalent;. Furthermore, surveillance programs can allow the detection of local differences in prevalence of HPV genotypes not covered by the current vaccines, such as HPV-51 in North Sardinia.

## Conclusions

To our knowledge, this is the first survey addressing the prevalence of HPV infection in Northern Sardinian population and it draws attention to the unusual high prevalence of genotype HPV 51 in this territory. Even if some studies in other regions have already described a high frequency of this HR HPV type, HPV 51 continues to remain a rare genotype almost everywhere. Given the recent implementation in Italy of a widespread vaccination program with prophylactic HPV vaccines not containing HPV 51, it is important to emphasize the high prevalence of this "atypical" HPV genotype from the start of the vaccination campaign, in order to avoid attributing to the vaccination program in the future a possible selection effect with consequent HPV replacement. Therefore, our results confirm the need of pre and post-vaccine extensive surveillance in order to better understand the effectiveness of vaccination.

However, the precision of our study, as displayed by the wide confidence intervals, may be limited by the small sample size. Therefore, for some low prevalent genotypes the statistical power was low and the possibility that the true prevalence may be different than the observed one cannot be excluded. Moreover, we know that the results of the study may be limited because of the characteristics of the screening adopted in Northern Sardinia so far. In fact, a voluntary based access instead of a systematic one may have selected those patients with higher prevalence of symptoms and it may explain the higher HPV prevalence observed. However, the potential selection bias might be not so influent, since no significant differences between positive and negative women were detected in terms of compliance to the PAP test in the last 3 years, as reported in Tab. 1. Therefore, this study should be able to give a reliable picture of the epidemiological HPV type pattern of infection, at the beginning of the vaccination campaign in the local population.

In conclusion, this study has confirmed the high prevalence of HPV infection in our region, as previously reported for Southern Sardinia, moreover highlighted the possible role of uncommon genotypes such as HPV-51.

## List of abbreviations

HPVs: Human papillomaviruses; HR-HPV: High Risk - Human papillomaviruses; LR-HPV: Low Risk - Human papillomaviruses; dNTPs: Deoxynucleotide triphosphates; ASCUS: Atypical Cells of Undetermined Significance; LSIL: Low-grade of Squamous Intraepithelial Lesion; HSIL: High-grade of Squamous Intraepithelial Lesion; OR: Odds ratio; CI: Confidence Intervals; CIN: Cervical Intraepitelial Neoplasia; PAP test: Papanicolau test

## Competing interests

The authors declares that he has no competing interests.

## Authors' contributions

All of the authors participated in planning and design of the study, and all read and approved the manuscript. AP, SP and CC participated to define protocols for molecular analysis. SD and GC selected the patients; VM carried out the histological examination. GS and PC performed the statistical analysis.

AP, GS, PC and EM conceived the study, participated in its design and wrote the manuscript.

## Pre-publication history

The pre-publication history for this paper can be accessed here:

http://www.biomedcentral.com/1471-2458/11/785/prepub
